# Insulin Autoimmune Syndrome: A Systematic Review

**DOI:** 10.1155/2023/1225676

**Published:** 2023-02-15

**Authors:** MingXu Lin, YuHua Chen, Jie Ning

**Affiliations:** ^1^Department of Endocrinology, The Affiliated Central Hospital of Shenzhen Longhua District, Guangdong Medical University, Shenzhen 518110, Guangdong, China; ^2^Guangdong Medical University, Zhanjiang 524000, Guangdong, China; ^3^Department of Endocrinology, Longgang District People's Hospital of Shenzhen, Shenzhen 518172, Guangdong, China

## Abstract

Insulin autoimmune syndrome (IAS) is a rare endocrine disorder characterized by recurrent episodes of severe hypoglycemia, markedly elevated serum insulin, and positive insulin autoantibodies. In recent years, various countries have reported it one after another. It can be seen that we must pay attention to this disease. The diagnosis of IAS is challenging, requiring a careful workup aimed at excluding other causes of hyperinsulinemic hypoglycemia. High levels of insulin autoantibodies are found in patients, and C-peptide is not parallel to insulin, which could be diagnostic. IAS is a self-limiting disease with a good prognosis. Its treatment mainly includes symptomatic supportive treatment, such as adjusting the diet and using acarbose and other drugs to delay the absorption of glucose to prevent hypoglycemia. For patients with severe symptoms, available treatments may include drugs that reduce pancreatic insulin secretion (such as somatostatin and diazoxide), immunosuppressants (glucocorticoids, zaprin, and rituximab), and even plasma exchange to remove autoantibodies from the body. This review provides a comprehensive analysis of the epidemiology, pathogenesis, clinical manifestations, diagnosis and identification, and monitoring and treatment management of IAS.

## 1. Introduction

IAS, also known as Hirata disease, was first described by Hirata in Japan in 1970 [1]. IAS is characterized by recurrent spontaneous hypoglycemia, the presence of insulin autoantibodies in the body, significantly elevated serum insulin, and no history of exogenous insulin injections. However, in recent years, it has been found that insulin and its analogs can also induce IAS, and the concept of nonclassical IAS has been proposed [2], so we need to update this view.

There is no clear accepted incidence, and current data are based on a comprehensive analysis of case reports. The age of onset of IAS patients varies greatly. There are literature statistics about IAS [3]. From 1994 to May 2017, 142 cases of IAS were reported in the Chinese biomedical literature database, including 62 males and 80 females aged 9–82 years old, with a median age of 46. Among these, 18 were idiopathic IAS. The results showed that methimazole treatment of Graves' disease was the most common cause of IAS. Other causative drugs included tiopronin, captopril, insulin, d-penicillamine, propranolol, and alpha-sulfur bitter.

IAS is a disease closely related to HLA, specifically to HLA-DRB1*∗*0406, DQB1*∗*0302, DQA1*∗*0301, DRB1*∗*0415 [4], DRB1*∗*13: 01 [5], and HLA-DRB1*∗*0406, which are most frequently associated with IAS. The DRB1*∗*0406 genotype is more frequent in East Asian patients, including the Japanese, than in Western populations, being rare in Caucasians, which explains why IAS is more common in East Asians and less common in Caucasians. Most Asian patients with IAS have DRB1*∗*0406, whereas DRB1*∗*0403 occurs in affected Caucasians, which may explain the difference in incidence between these two ethnic groups [6]. It has been reported that Caucasians with the HLA-DRB1*∗*0403 allele can develop IAS associated with lipoic acid, indicating that this allele is genetically susceptible to IAS in Caucasians and possibly other populations [6].

Approximately 80% of IAS cases coexist with other autoimmune diseases, namely, most patients with Graves' disease [7] and others with systemic lupus erythematosus [8], rheumatoid arthritis, chronic hepatitis. Other less common coexisting diseases include ankylosing spondylitis [9], antineutrophil cytoplasmic antibody-associated glomerulonephritis, alcoholic cirrhosis, polymyositis and systemic sclerosis, psoriasis [10], multiple myeloma [11, 12], and monoclonal gammopathy [13].

This review summarizes the number of reported cases of IAS, drugs related to the occurrence of IAS, coexisting diseases, and treatment schemes reported for IAS since IAS was first described in 1970 (Tables 1–3).

## 2. Materials and Methods

We searched PubMed, China National Knowledge Infrastructure, Wan-fang Data Knowledge Service Platform, and China Science and Technology Journal Database to identify studies and reviews published between January 1, 1970, and December 31, 2022, relevant to the scope of this Seminar with the terms “insulin autoimmune syndrome,” “IAS,” “Hirata disease,” “insulin autoantibody,” “hyperinsulinemic hypoglycemia,” “immunoreactive insulin,” and “autoimmune hypoglycemia.”

Articles were considered regardless of language. We selected references that provided current, evidence-based insight into insulin autoimmune syndrome. Most of the articles selected were published within the past 5 years, although we also included highly referenced, older publications that contributed to new knowledge or understanding of insulin autoimmune syndrome.

## 3. Results

### 3.1. Classification of IAS

IAS can be classified according to different perspectives. According to the history of exogenous insulin injection, insulin syndrome is divided into classic IAS and nonclassical IAS [2]. According to the original definition of IAS, it only occurs in patients without a history of exogenous insulin injection; however, an association with the use of exogenous insulin or insulin analogs has been identified in recent years [14, 15]. Individuals who use insulin glulisine (such as glargine) can have symptoms similar to IAS, usually manifesting as postprandial hypoglycemia, obvious neurological symptoms and changes in their state of consciousness, accompanied by high titers of insulin autoantibodies and serum hyperinsulinemia, so the concept of nonclassical IAS was proposed to distinguish it from the previous classic IAS.

According to the presence or absence of drug induction, IAS can be divided into drug-induced and idiopathic (primary) IAS. As the name suggests, drug-induced IAS patients have a history of taking insulin-related autoimmune syndrome drugs before onset. The most common such drugs are methimazole [16, 17] and lipoic acid [18–22]. Others include clopidogrel [23–26], proton pump inhibitors [27] (pantoprazole [8], omeprazole), carbimazole [28], coenzyme Q10 [29], antituberculosis drugs [30], gliclazide [31], captopril [32], loxoprofen sodium [33], and albumin preparations [34], within the preceding week. Studies have shown that approximately half of IAS patients have a history of medication before the onset of the disease, and more than 90% of them used drugs containing sulfhydryl groups such as *α*-lipoic acid or its metabolites [35]. However, among patients without a history of taking drugs related to IAS, some scholars have identified their condition as idiopathic or primary IAS [36], manifesting as unexplained IAS.

### 3.2. The Pathogenesis and Pathophysiology of IAS

It has been reported that more than 50% of patients diagnosed with IAS have previously received drugs containing sulfhydryl groups, which are thought to be related to the chemical and immune responses of insulin molecules and can trigger the production of insulin autoantibodies [37]. Thiol-containing drugs, such as lipoic acid, are a widely used nutritional supplement, taken as an adjunct treatment for diabetic neuropathy and other conditions. Lipoic acid can also increase insulin sensitivity and improve glycemic control in various clinical situations, but it can promote the dissociation of the S-S bond of insulin, expose the peptide to antigen-presenting cells, and stimulate T cells in IAS patients; thus, insulin autoantibodies are formed. Insulin autoantibodies produced in IAS patients are different from those caused by exogenous insulin. Insulin antibody (IA) is commonly found in patients receiving insulin treatment. Insulin antibody (IA) usually has low binding capacity and high affinity and rarely causes hypoglycemia, which is different from the high binding capacity and low affinity of IAA in IAS [38]. Approximately 40% of diabetic patients receiving human insulin injections have positive serum insulin antibodies, but these antibodies have little effect on blood glucose [2]. Scatchard analysis showed that insulin autoantibodies in IAS patients have a significantly lower affinity constant for insulin and a much stronger binding capacity, showing low affinity/high binding capacity, so autoantibodies could easily bind to insulin [39]. Scatchard analysis of IAS reported that insulin antibodies are polyclonal with a dissociation constant at high-affinity sites that are significantly lower than those produced by injected insulin [40].

**Figure 1:**
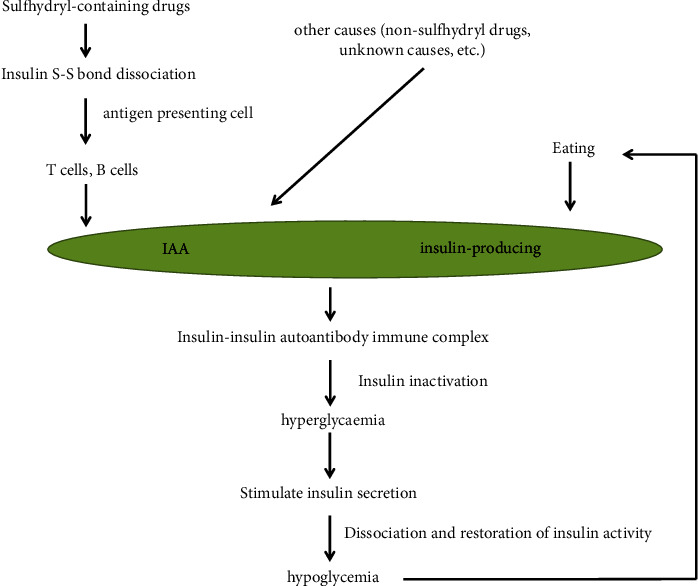
Pathogenesis of IAS.

In Japan, the majority of IAS patients have IgG polyclonal IAAs, whereas IAAs are monoclonal in more than half of reported cases of IAS in non-Asian patients. Although the most common IAA immunoglobulin class is IgG, IgA and IgM have also been reported in IAA cases [41]. Once plasma insulin (antigen) increases to a certain level, the insulin-autoantibody complex becomes unstable and abruptly disintegrates, leading to postprandial hypoglycemia. In addition, some drugs that do not contain sulfhydryl groups, such as clopidogrel, themselves do not contain sulfhydryl groups but become biologically active compounds after being metabolized by cytochrome P450 enzymes, and the active metabolites contain sulfhydryl groups, which can cause IAS. The mechanism is similar to that of sulfhydryl-containing drugs (Figure 1).

For IAS caused by insulin analogs, the mechanism is not clear, but it also works through IAA. The amino acid sequence of insulin analogs is different from that of human insulin and can be considered nonself-antigens, especially zinc-mediated insulin, because it is located at the injection site, with a longer residence time and easier uptake by antigen-presenting cells. The zinc-free insulin analogs are rapidly absorbed into the blood after subcutaneous injection, so they are considered to be less immunogenic than other insulins. Drugs containing sulfhydryl groups or metabolized sulfhydryl groups, as well as insulin analogs, are believed to be immunogenic and lead to the production of insulin autoantibodies. As a matter of fact, even though modern insulin analogs have a low immunogenicity, insulin antibodies may sometimes be detected in patients receiving insulin therapy, but these antibodies are rarely capable of causing hyperglycemia or hypoglycemia. This is because the insulin antibodies that develop following exposure to exogenous insulin are more often characterized by a higher affinity and a lower binding capacity against insulin compared to IAA. As a consequence, they mostly result in smaller antigen-antibody complexes which have a lower spontaneous dissociation rate, and thus, they are unable to produce significant glycemic fluctuations. Nevertheless, insulin antibodies developing after the administration of exogenous insulin may seldom present with features similar to those of IAA, such as a high binding capacity and low affinity, thus resulting in glycemic instability [42]. However, some drugs that do not contain sulfhydryl groups, such as loxoprofen sodium, can also lead to the production of insulin autoantibodies, but there is no relevant literature on the possible mechanism.

In patients with this low-affinity/high-binding antibody, food-stimulated pancreatic insulin rapidly binds to insulin autoantibodies after eating, and this binding reduces insulin utilization by receptors in the liver and peripheral tissues, masking the biological activity of insulin and leading to postprandial hyperglycemia, which further stimulates insulin secretion from the pancreas [43]. Dozio et al.[43] investigated the mechanism of hypoglycemia by performing 125I-labeled insulin scintigraphy in a patient with IAS. Following intravenous administration of 125I-labeled insulin, the patient showed sustained labeled insulin activity in the blood, with no uptake of labeled hormones in the liver or kidneys. In the late postprandial period, due to the low affinity of insulin autoantibodies to insulin, excess insulin does not bind to the insulin autoantibodies, and the free insulin exerts its inherent hypoglycemic effect, resulting in a sharp drop in blood glucose and late postprandial hypoglycemia.

In addition, some patients mainly present with fasting or early morning hypoglycemia, which is caused by the separation of insulin and insulin antibodies due to changes in the internal environment during the night (which may be related to decreased insulin antibody affinity). Insulin antibodies become saturated with insulin during the day, which means that fewer antibodies are available to bind to insulin in the middle of the night [2].

### 3.3. Clinical Manifestations of IAS

Hypoglycemia or alternating episodes of hypoglycemia and hyperglycemia: patients often show the typical Whipple triad, that is, 1. symptoms and signs of hypoglycemia, including autonomic hypoglycemia symptoms (tremor, palpitation, anxiety, sweating, hunger, and sensory abnormalities, which are largely caused by sympathetic activation) and cerebral neuronal hypoglycemia symptoms (mainly including cognitive impairment, behavioral changes, psychomotor abnormalities, and seizures and coma when their blood glucose concentration is lower), 2. blood glucose <2.8 mmol/l during an attack, and 3. the symptoms will be relieved rapidly after administering glucose-increasing drugs. According to previous case reports, approximately 42% of patients showed postprandial delayed hypoglycemia, 31% had fasting hypoglycemia, and 24% had both postprandial and fasting hypoglycemia [44, 45]. As mentioned previously, the occurrence of hyperglycemia and postprandial delayed hypoglycemia is mainly caused by the binding and dissociation of insulin autoantibodies and insulin. Continuous blood glucose monitoring system data confirmed that IAS alternates between postprandial hyperglycemia and postprandial hypoglycemia [46]. A few cases of insulin autoimmunity manifested as hypoglycemia after repeated diabetic ketoacidosis [47]. In addition, some patients with recurrent hypoglycemia and a long course of the disease may gain weight due to frequent eating [48, 49].

### 3.4. IAS-Related Tests

At present, the most commonly used test for diagnosing IAS is IAA, but a commercial kit for detecting IAA has not been widely used [49]. Insulin autoantibodies in IAS patients are usually IgG class immunoglobulins, but IAA can also belong to other classes (such as IgA and IgM). The kits on the market usually only recognize the IgG class, so they sometimes give false negative results; thus, a negative IAA test does not completely rule out IAS. To avoid misleading results, it is critical that IAA assays detect all immunoglobulin classes and subclasses [41].

Uchigata et al. [25] suggested that the measurement of insulin may cause erroneous results due to the interference of antibodies with the measurement results. By adding PEG or ultrafiltration, if the insulin is combined with antibodies, the heavy protein molecules are precipitated, and free insulin can be measured in the supernatant. Serum-free insulin levels are detected after polyethylene glycol precipitation of IAA, and the free insulin is separated from antibody-bound insulin; in suspected cases of IAS, a significant difference between total serum insulin and free insulin strongly suggests IAS [20].

Basu and John [50] reported the cases of 7 white IAS patients and found that during hypoglycemia, the concentrations of insulin, proinsulin, and C-peptide greatly exceeded those observed in insulinoma patients, but they were falsely elevated due to the interference of autoantibodies. This confirmed the hypothesis that autoantibody interference in immunoassays leads to excessive insulin concentrations by measuring free insulin after a mixed meal test.

Polyethylene glycol precipitation and gel chromatography separation methods can be used to identify whether there are macromolecular immune complexes bound to insulins, especially for patients with negative insulin autoantibodies. Polyethylene glycol can precipitate all immunoglobulin IAA [41], but it has little effect on small molecular proteins such as insulin. If there is an insulin-insulin autoantibody immune complex in the serum, it will be precipitated by polyethylene glycol, and the insulin level measured after precipitation will decrease. Gel chromatography can separate proteins according to their molecular weight, and macromolecular insulin-insulin autoantibody complexes will be eluted before the smaller molecules of free insulin, showing high levels of “insulin” (actually insulin-insulin autoantibody complexes) [51]. The polyethylene glycol precipitation method is an inexpensive laboratory method that can be achieved by virtually any laboratory, with the advantages of a simple operation, short time consumption, and low cost, and it can be used as a primary screening experiment. However, it should be noted that the polyethylene glycol method is susceptible to the interference of macromolecular proteins and temperature, which affects its specificity. The gel chromatography separation method is relatively complicated, time-consuming, and expensive (it needs to measure insulin and adiponectin in multiple collection tubes), so it is not suitable for primary screening. Conditional laboratories can use this method as a confirmatory test [41].

Insulin and C-peptide are cosecreted into the portal circulatory system in an equimolar ratio by pancreatic beta cells. Insulin is mainly cleared by the liver, C-peptide is mainly metabolized by the kidney, and the metabolism of C-peptide is significantly slower, so the half-lives of these two are 5–10 minutes and 30–35 minutes, respectively. Thus, the molar ratio of insulin to C-peptide is generally less than 1, although insulin and C-peptide are secreted in equal proportions. In general, there are two situations that result in a molar ratio of insulin to C-peptide greater than 1. One is in patients with IAS. Second, during artificial hypoglycemia caused by exogenous insulin, the insulin concentration increases, and the C-peptide level is inhibited so that the ratio is greater than 1 [52]. However, a study that reviewed 16 patients with IAS pointed out the limitations of the insulin to C-peptide molar ratio; 9 of the 16 patients had an insulin to C-peptide molar ratio >1, while 6 patients had insulin to C-peptide molar ratio. The molar ratio to C-peptide is < 1, which indicates that the molar ratio of insulin to C-peptide can only be used for the auxiliary diagnosis of IAS [53] (Table 4).

It is generally believed that in patients with IAS, the typical monophasic pattern of hyperinsulinemia measured by the oral glucose tolerance test (OGTT) of insulin secretion kinetics is evident; however, there are also some patients whose insulin secretion kinetics by the OGTT are shown as biphasic insulin secretion kinetics distinct from typical IAS. One study reported a pair of sisters who both suffered from IAS, and their insulin secretion kinetics measured by the OGTT showed a biphasic insulin secretion kinetic pattern [40]. Compared with 32 healthy adults, IAS patients had significantly lower fasting blood glucose levels than controls. However, the oral glucose tolerance test (OGTT) showed that all patients with IAS had impaired glucose tolerance or diabetes, so their OGTT 2 h blood glucose level was significantly higher than that of the control group [45]. On the OGTT, some patients showed a diabetic pattern, while others showed impaired glucose tolerance [54].

Hyperglycated hemoglobin levels are common in patients with IAS, but there are some patients whose glycated hemoglobin is within the normal range, and the detection of glycated hemoglobin is of little significance for the diagnosis of IAS [41].

Some studies have analyzed the clinical indicators of IAS patients and normal healthy subjects [54]: Glycated albumin (GA) levels and GA/HbA1c ratios in IAS patients were significantly higher than those in the control group, but there was no significant difference in HbA1c levels between the two groups. In the case of spontaneous remission of IAS, there was a significant correlation between anti-insulin antibodies and GA but not HbA1c. With the improvement of clinical symptoms, the anti-insulin antibody, GA, and GA/HbA1c ratio of 3 IAS patients decreased, but HbA1c did not change significantly. These results suggest that GA and the GA/HbA1c ratio are useful indicators for determining the level of disease activity in patients with IAS. Glycated albumin (GA), on the other hand, is an intermediate indicator of glycemic control, reflecting fluctuations in blood sugar, as well as average blood sugar levels. In addition, the GA/HbA1c ratio is an indicator reflecting blood sugar fluctuations. Through continuous glucose monitoring (CGM) analysis, it has been demonstrated that patients with IAS have increased glycemic excursions as they develop postprandial hyperglycemia in addition to fasting hypoglycemia.

The purpose of the 72-hour fasting test is to provoke hypoglycemia in the absence of food. Normal individuals do not develop symptomatic hypoglycemia after prolonged fasting due to hormone-mediated increases in glucose production. Prolonged fasting can lead to hypoglycemia only when there is a deficit in the ability to maintain normoglycemia, such as too much insulin. The 72-hour fasting test is a standard test for the diagnosis of insulinoma [55] and can be used to differentiate insulinoma from IAS. In general, the IAS 72-hour fasting test is generally free of hypoglycemia, and there are case reports of no spontaneous hypoglycemia in patients undergoing the 72-hour fasting test. However, after the 72-hour fasting test, reactive symptomatic hypoglycemia occurred 12 hours after eating. Some patients with IAS may also have hypoglycemia during the 72-hour fasting test, so the 72-hour fasting test cannot completely distinguish IAS from insulinoma. One study analyzed the 72-hour fasting test of 74 insulinomas, and all patients with insulinoma developed hypoglycemia [52].

A continuous glucose monitoring system continuously converts the glucose level present in the interstitial tissue into an electrical signal whose intensity is proportional to the glucose content. The sensor measures the tissue fluid glucose concentration every 5 minutes on average. Continuous glucose monitoring systems can effectively monitor diet and treatment measures [53]. Continuous blood glucose testing showed that blood glucose levels worsened after glucocorticoid treatment, and the hyperglycemic state associated with hypoglycemia persisted longer. Glucosidase inhibitors and plasma exchange are more effective in limiting glucose intake than glucocorticoids or diet alone. The use of continuous glucose monitoring systems has also demonstrated the effectiveness of plasma exchange in treating insulin autoimmune symptoms by normalizing blood glucose and antibody levels, although it is not a curative treatment. The use of continuous glucose monitoring systems provides a way to precisely assess the efficacy of different drugs [56].

Notably, some patients are positive for antibodies, anti-dsDNA, and rheumatoid factor, but whether they are associated with IAS requires further investigation [43].

### 3.5. Differential Diagnosis

IAS needs to be distinguished from insulinoma. In patients with insulinoma, hypoglycemia often occurs in the fasting state, while in IAS, it often occurs repeatedly at night and in the early morning. A fasting test is a method of differential diagnosis. The insulin concentration level of insulinoma is generally less than 1000 mu/ml, and the increase in insulin concentration is synchronized with the increase in C-peptide levels, while the insulin concentration of IAS patients is often greater than 1000 mu/ml [3]. The blood glucose level of IAS fluctuates widely. Hypoglycemia and hyperglycemia can occur alternately, while insulinoma generally does not have hyperglycemia. IAA is the key to differentiation. Most IAS is positive, and a few are negative, but the IAA level of insulinoma is always negative. Abdominal computed tomography (CT) is also an important examination method to distinguish IAS and insulinoma. Generally, CT is not abnormal in patients with IAS, while patients with insulinoma often have positive findings. However, it should be noted that a small number of IAS patients are complicated with pancreatic space-occupying lesions, which are difficult to distinguish and require special attention. A correct diagnosis is very important because it can prevent patients with hypoglycemia from undergoing unnecessary pancreatic surgery.

IAS must also be distinguished from hypoglycemia caused by insulin receptor antibodies, also known as type B insulin resistance. In these cases, antibodies cause hypoglycemia through the direct activation of insulin receptors. It is characterized by obvious acanthosis nigricans, accompanied by severe hyperglycemia and other autoimmune diseases [57].

IAS also needs to be distinguished from hypoglycemia caused by hypoglycemic drugs or insulin and its analogs. In diabetic patients (type 1 and type 2 diabetes), IAS can also occur. Therefore, it is particularly important to identify hypoglycemia caused by these two causes. Another differential diagnosis of IAS is hypoglycemia caused by advanced dumping syndrome [58].

For patients with hypoglycemia and a history of taking drugs that induce IAS, IAS should be considered as a differential diagnosis. High concentrations of autoantibodies, C-peptide, and insulin imbalance are highly suspicious for IAS.

The key points of IAS differential diagnosis are as follows (Table 5):

### 3.6. Treatment

At present, the treatment plan for insulin syndrome has not been unified, and various treatment plans have been proposed in various studies, including drug withdrawal, frequent small meals, avoidance of monosaccharide intake, glucocorticoid treatment [59, 60], plasma exchange [61], immunosuppressive agents (such as sulfur azathioprine [56] and 6-mercaptopurine), and rituximab [56, 61, 62] (Table 6).

It is mentioned in the literature that the insulin autoantibodies will disappear on their own within a few months to several years, which is one of the pieces of evidence that IAS is self-limiting. Hirata and Uchigata reported that most hypoglycemia lasts less than 1 month, with an average of 3–6 months [54]. A study found that among 73 patients with IAS, 5 patients had mild hypoglycemia that lasted for more than 1 year. Some patients can avoid or reduce the occurrence of hypoglycemia symptoms simply by discontinuing IAS-related drugs or discontinuing other related drugs, eating small meals, and avoiding monosaccharide intake. This is the first-line treatment for IAS. It has been reported that although the hypoglycemia symptoms disappear after a few weeks or months, the antibodies may persist for several years, and thus, the mechanism behind the disappearance or significant reduction of hypoglycemia is still unclear. In some obese patients with IAS, the improvement in hypoglycemic episodes with weight loss may be due to a reduction in insulin resistance resulting in a lower postprandial insulin peak [63].

An additional treatment strategy for IASs is to buffer the effects of autoantibodies that bind to endogenous insulin and reduce the formation of complexes with endogenous insulin. Acarbose and voglibose have inhibitory effects on intestinal *α*-glucosidase; they can delay the absorption of postprandial carbohydrates, make the blood sugar rise more slowly, reduce the stimulation of blood sugar to the pancreas, and trigger the production of insulin. The secretion of insulin decreases, avoiding the combination of a large amount of insulin with insulin autoantibodies, and improves the symptoms of postprandial hypoglycemia in patients with the autoimmune syndrome. In addition, a study [64] showed that cornstarch delays absorption and has been used to treat glycogen storage disorders in patients with hypoglycemia and unstable glycemic control in patients with type 1 diabetes. According to this theoretical basis, IAS is treated by dietary management with cornstarch. After cornstarch is given, the symptoms of hypoglycemia at night disappear, indicating that cornstarch treatment is effective, and its mechanism is similar to that of *α*-glucosidase inhibitors to delay carbohydrate absorption. However, this is the first and only case of cornstarch therapy for the treatment of IAS, and the possibility of spontaneous remission cannot be completely ruled out, but it suggests the potential usefulness of cornstarch therapy in preventing hypoglycemia in patients with IAS.

Glucocorticoid therapy can be used for patients with IAS that cannot be controlled by discontinuation of IAS-related drugs, dietary management, and the use of alpha-glucosidase inhibitors. Many studies have mentioned that in cases where the above two methods cannot relieve the hypoglycemia, the use of glucocorticoids can effectively reduce the frequency and severity of hypoglycemia, and it has a good effect in reducing insulin autoantibodies in patients. The most commonly used glucocorticoid is methylprednisolone. A study in Japan [57] showed that corticosteroid treatment reduced the number of insulin receptor binding sites and avoided hypoglycemic episodes, suggesting the effectiveness of corticosteroids in the treatment of IAS.

Among other second-line regimens, immunosuppressants (azathioprine and 6-mercaptopurine), monoclonal antibodies (rituximab), plasma exchange, and strategies to reduce insulin release (somatostatin analogs, pancreatectomy) have shown some efficacy in the management of IAS. Rituximab reduces IAAs and improves hypoglycemia.

Partial pancreatectomy may be feasible in refractory patients with frequent episodes of hypoglycemia [53] but is currently rarely used in clinical practice. In a patient diagnosed with IAS, the patient developed metabolic acidosis, and the symptoms of hypoglycemia disappeared after the administration of sodium bicarbonate, but its mechanism and efficacy still need to be confirmed by a large number of studies.

IAS is mostly a self-limiting disease with a good prognosis. In the absence of other serious complications, after removing the triggers and using immunosuppressive drugs or drugs that delay glucose absorption, the symptoms mostly disappear after a few months, and the outcome is good.

## 4. Conclusion

IAS is a rare endocrine disease, but in Japan, IAS is the third leading cause of spontaneous hypoglycemia, and the incidence of this disease is increasing. An increasing number of countries has reported this disease one after another. Its increasing incidence is not just due to improvements in detection methods; the widespread use of drugs is also a factor that cannot be ignored. The current drug spectrum related to IAS is expanding, which requires us to pay more attention to it. For this disease, insufficient laboratory examinations and other auxiliary examinations can easily cause misdiagnosis and missed diagnosis, which suggests that we need to develop more effective detection methods. There is no standardization of the treatment plan at present, and additional research is needed to unify and develop an effective treatment plan. These are problems that urgently need to be solved.

## Figures and Tables

**Table 1:** Countries reporting IAS and the number of cases (*N*).

Countries	*N*
Japan	350 (44.02%)
China	330 (41.51%)
South Korea	13 (1.64%)
India	11 (1.38%)
Türkiye	3 (0.38%)
Italy	10 (1.26%)
Canada	1 (0.13%)
Switzerland	2 (0.25%)
Israel	1 (0.13%)
Australia	3 (0.38%)
Nepal	1 (0.13%)
U.S.A	34 (4.28%)
Brazil	4 (0.50%)
Kuwait	1 (0.13%)
Germany	4 (0.50%)
Argentina	2 (0.25%)
Netherlands	2 (0.25%)
Russia	1 (0.13%)
Poland	2 (0.25%)
Thailand	1 (0.13%)
UK	6 (0.75%)
France	4 (0.50%)
Spain	4 (0.50%)
Chile	2 (0.25%)
New Zealand	1 (0.13%)
Portugal	2 (0.25%)
Total	795

**Table 2:** IAS-related drugs and the number of cases (*N*).

Related drugs	*N*
Methimazole	170 (21.38%)
Alpha lipoic acid	51 (6.42%)
Clopidogrel	8 (1.01%)
Esomeprazole	1 (0.13%)
Captopril	8 (1.01%)
Insulin and its analogues	20 (2.52%)
Sulfhydryl-containing health products	2 (0.25%)
Insulin aspartate 30	27 (3.40%)
Insulin glargine	2 (0.25%)
Glucosamine sulfate-containing drugs	1 (0.13%)
Insulin lispro	3 (0.38%)
131I therapy	2 (0.25%)
Mercaptan	2 (0.25%)
Tiopronin	54 (6.80%)
Zinc protamine recombinant human insulin	11 (1.38%)
Acetylcysteine	1 (0.13%)
Penicillamine	2 (0.25%)
Carbimazole	3 (0.38%)
Propylthiouracil	3 (0.38%)
Penicillin G	1 (0.13%)
Ganshulin 50R	2 (0.25%)
Coenzyme Q10	1 (0.13%)
Omeprazole	1 (0.13%)
Human serum albumin	1 (0.13%)
Antituberculosis treatment	2 (0.25%)
Loxoprofen sodium	2 (0.25%)
Insulin glulisine	1 (0.13%)
Pantoprazole	1 (0.13%)
Methionine-containing supplements	1 (0.13%)
Imipenem	1 (0.13%)
Azathioprine	1 (0.13%)
Glimepiride	1 (0.13%)
Glutathione	8 (1.01%)
Gold thioglucoce	1 (0.13%)
Acegratone	1 (0.13%)
Interferon-alfa	1 (0.13%)
Gliclazide	1 (0.13%)
Unknown or no	399 (50.19%)
Total	795

**Table 3:** IAS-related autoimmune diseases and the number of cases (*N*).

Autoimmune diseases	*N*
Graves	190 (23.90%)
Latent autoimmune diabetes in adults	2 (0.25%)
Systemic lupus erythematosus	7 (0.88%)
Hashimoto thyroiditis	15 (1.89%)
Sjogren's syndrome	2 (0.25%)
Chronic nephritis syndrome	1 (0.13%)
Circumscribed scleroderma	1 (0.13%)
Rheumatoid arthritis	10 (1.26%)
Thrombocytopenic purpura	1 (0.13%)
Acanthosis nigricans	2 (0.25%)
Systemic scleroderma	2 (0.25%)
Psoriasis	2 (0.25%)
Myelodysplastic syndrome	1 (0.13%)
Multiple myeloma	8 (1.01%)
Ankylosing spondylitis	2 (0.25%)
Membranous proliferative glomerulonephritis	1 (0.13%)
Autoimmune polyglandular syndrome type 3	1 (0.13%)
Hypothyroidism	7 (0.88%)
Antiphospholipid syndrome	1 (0.13%)
ANCA associated nephritis	1 (0.13%)
Interstitial lung disease	1 (0.13%)
Monoclonal gammaglobulosis	4 (0.50%)
Nephrotic syndrome	1 (0.13%)
Leukoderma	2 (0.25%)
Polymyositis	1 (0.13%)
Unknown or no	530 (66.67%)
Total	795

**Table 4:** IAS-related detection and its advantages and shortcoming.

Tests	Advantages	Shortcomings
IAA	Common indicators, high detection rate	Not widely used yet; the kits on the market can only detect IgG IAA generally
Polyethylene glycol precipitation	IAA can be precipitated	Easy to be disturbed by macromolecular proteins and temperature
Gel filtration chromatography	It is more specific than the above two methods	Complex operation, high requirements for the laboratory, long time-consuming and expensive
Insulin/C-peptide molar ratio	It can assist diagnosis of IAS	It can be affected by exogenous insulin
HLA gene detection	Identify the pathogenic gene locus	Few domestic and foreign activities, high cost

**Table 5:** The key points of IAS differential diagnosis.

Differentiation point/disease	IAS	Insulinoma	Type B insulin resistance
High-risk population	East Asian people (especially Japanese)	Unknown	Black women
Insulin	>1000 mU/L	<1000 mU/L	Elevated
Insulin/C-peptide molar ratio	>1	<1	<1
IAA	Positive	Negative	Negative
Insulin recovery following precipitation with PEG	Low (5–10%)	Normal (>70%)	Normal (>70%)
Abdominal CT	Generally negative	Space occupying lesion	Generally negative

**Table 6:** Treatment and reported cases of IAS.

Treatment	*N*
Stop relevant drugs	54
Adjust diet	67
Methylprednisolone	7
Acarbose	59
Acarbose + prednisone	23
Prednisone	116
Traditional Chinese medicine	2
Voglibose	6
Hydrocortisone + prednisone	1
Plasma exchange + methylprednisolone injection + oral methylprednisolone	2
Methylprednisolone + prednisone	2
Methylprednisolone + prednisone + acarbose	1
Prednisone + traditional Chinese medicine	1
Hydrocortisone	6
Methylprednisolone + acarbose	1
Pancreatic surgery	6
Prednisone + methylprednisolone + plasma exchange	1
Diazoxide	3
Dexamethasone + diazoxide + plasma exchange	1
Voglibose + prednisone	1
Sodium bicarbonate	1
Prednisone + acarbose + rituximab	1
Plasma exchange	1
Prednisone + rituximab	2
Methylprednisolone + rituximab	1
Prednisone + dexamethasone + plasma exchange + rituximab	1
Dexamethasone	1
Rituximab	1
Glucocorticoid + ɑ-glucosidase inhibitor + plasma exchange	1
Prednisone + plasma exchange + rituximab	1
Acarbose + plasma exchange + rituximab	1
Cornstarch	1
Prednisone + dexamethasone	1
Prednisone + diazoxide	3
Prednisone + cyclophosphamide + steroid shock	1
Plasma exchange + prednisone + cyclophosphamide	1
Dexamethasone + acarbose	1

## Data Availability

The data that support the findings of this study can be obtained from the corresponding author upon reasonable request.

## Abbreviations

IAS: Insulin autoimmune syndrome
IAA: Insulin autoantibody
IA: Insulin antibody
CT: Computed tomography
OGTT: Oral glucose tolerance test
GA: Glycated albumin.

## References

[1] Y H. (1970). Insulin autoimmunity in a case of spontaneous hypoglycemia. *Journal of the Japan Diabetic Society*.

[2] Shen Y., Song X., Ren Y. (2019). Insulin autoimmune syndrome induced by exogenous insulin injection: a four-case series. *BMC Endocrine Disorders*.

[3] Zeng X. X., Tang Y. L., Hu K. X., Wang J., Zhu L. Y. (2017). Insulin autoimmune syndrome in a pregnant female: a rare case report Medicine (United States).

[4] Cambria V., Beccuti G., Gatti F., Bona C., Maccario M. (2020). Hla DRB1∗0415: a new possible genetic susceptibility factor for Hirata’s disease. *Endocrine*.

[5] Dos Santos T. J., Passone C. G. B., Ybarra M. (2019). Pitfalls in the diagnosis of insulin autoimmune syndrome (Hirata’s disease) in a hypoglycemic child: a case report and review of the literature. *Journal of Pediatric Endocrinology & Metabolism*.

[6] Yang C. C., Gu W. J., Lyu Z. H., Cheng Y., Jia A. H. (2021). One case report of insulin autoimmune syndrome induced by clopidogrel. *Zhonghua Nei Ke Za Zhi*.

[7] Wu H. Y., Chen I. H., Lee M. Y. (2022). Case report: hypoglycemia secondary to methimazole-induced insulin autoimmune syndrome in young Taiwanese woman with Graves’ disease. *Medicine (Baltimore)*.

[8] Ravindra S., Shetty S. (2020). Insulin autoimmune syndrome: a rare cause of spontaneous hypoglycaemia in non-diabetic individuals. *BMJ Case Reports*.

[9] Raizada N., Rahaman S. H., Kandasamy D., Jyotsna V. P. (2015). Rare association of insulin autoimmune syndrome with ankylosing spondylitis. *Endocrinol Diabetes Metab Case Rep*.

[10] Hunter A., Graham U., Lindsay J. R. (2018). Insulin Autoimmune Syndrome: a rare case of hypoglycaemia resolving with immunosuppression. *Ulster Medical Journal*.

[11] Ito H., Miyake T., Nakashima K., Ito Y., Tanahashi C., Uchigata Y. (2016). Insulin autoimmune syndrome accompanied by multiple myeloma. *Internal Medicine*.

[12] Yukina M., Nuralieva N., Solovyev M., Troshina E., Vasilyev E. (2020). Insulin autoimmune syndrome. *Endocrinol Diabetes Metab Case Rep*.

[13] Solovyev M. V., Yukina M. Y., Troshina E. A. (2020). Hypoglycemic syndrome in patients with monoclonal gammopathy. *Problemy Endokrinologii*.

[14] Kobayashi S., Amano H., Kawaguchi Y., Yokoo T. (2019). A novel treatment of hyperinsulinemic hypoglycemia induced by insulin antibodies with alkali administration: a case report. *Journal of Medical Case Reports*.

[15] Chu J. P., Zheng X. W., Lu J. (2016). Insulin-induced autoimmune syndrome: a case report. *Experimental and Therapeutic Medicine*.

[16] Han R., Jiang X. (2020). Methimazole-induced insulin autoimmune syndrome in Graves’ disease with hypokalemia: a case report and literature review. *Experimental and Therapeutic Medicine*.

[17] Jain N., Savani M., Agarwal M., Kadaria D. (2016). Methimazole-induced insulin autoimmune syndrome. *Therapeutic Advances in Endocrinology and Metabolism*.

[18] Gullo D., Magliozzo M., Strano A., Piazza V. G., Stabile G. (2020). Insulin autoimmune syndrome misdiagnosed as an insulinoma in a woman presenting with a pancreatic cystic lesion and taking alpha lipoic acid: a lesson to be learned. *Hormones*.

[19] Moffa S., Improta I., Rocchetti S., Mezza T., Giaccari A. (2019). Potential cause-effect relationship between insulin autoimmune syndrome and alpha lipoic acid: two case reports. *Nutrition*.

[20] Veltroni A., Zambon G., Cingarlini S., Davì M. V. (2018). Autoimmune hypoglycaemia caused by alpha-lipoic acid: a rare condition in Caucasian patients. *Endocrinol Diabetes Metab Case Rep*.

[21] Izzo V., Greco C., Corradini D., Infante M., Staltari M. T. (2018). Insulin autoimmune syndrome in an Argentine woman taking *α*-lipoic acid: a case report and review of the literature. *SAGE Open Med Case Rep*.

[22] Maheshwari T. M., Sharma A., Maheshwari B. B. (2020). Insulin autoimmune syndrome: a rare cause of hypoglycemia. *Journal of Family Medicine and Primary Care*.

[23] Jiang Y., Wang L., Shi F., Zhou H., Zheng J. (2020). Insulin autoimmune syndrome after exposure to clopidogrel: a case report. *Endocrine, Metabolic &amp;amp; Immune Disorders: Drug Targets*.

[24] Calder G. L., Ward G. M., Sachithanandan N., MacIsaac R. J. (2020). Insulin autoimmune syndrome: a case of clopidogrel-induced autoimmune hypoglycemia. *Journal of Clinical Endocrinology and Metabolism*.

[25] Uchigata Y., Hirata Y. (1999). Insulin autoimmune syndrome (IAS, Hirata disease). *Ann Med Interne (Paris)*.

[26] Yamada E., Okada S., Saito T., Osaki A., Ozawa A., Yamada M. (2016). Insulin autoimmune syndrome during the administration of clopidogrel. *Journal of Diabetes*.

[27] Song L. Y., Li Y. J., Pang P., Xiao H. Y., Dou J. T. (2021). [Insulin autoimmune syndrome caused by proton pump inhibitors: a case report]. *Zhonghua Nei Ke Za Zhi*.

[28] Pant V., Bhandari B., Baral S., Bajracharya S. R. (2019). Insulin autoimmune syndrome as a cause of recurrent hypoglycemia in a carbimazole user: a case report from Nepal. *International Medical Case Reports Journal*.

[29] Kusano Y. (2019). Insulin autoimmune syndrome possibly caused by coenzyme Q10. *Journal of Rural Medicine*.

[30] Han J. S., Moon H. J., Kim J. S., Kim H. I., Kim C. H., Kim M. J. (2016). Anti-tuberculosistreatment-induced insulin autoimmune syndrome. *The Ewha Medical Journal*.

[31] Feng X., Yuan L., Hu Y., Zhu Y., Yang F. (2016). Gliclazide-induced insulin autoimmune syndrome: a rare case report and review on literature. *Endocrine, Metabolic &amp;amp; Immune Disorders: Drug Targets*.

[32] Reis M. Z. R., Fernandes V. O., Fontenele E. G. P., Sales A. P. A. M., Montenegro R. M., Quidute A. R. P. (2018). Insulin autoimmune syndrome in an Occidental woman: a case report and literature review. *Archives of Endocrinology and Metabolism*.

[33] Okazaki-Sakai S., Yoshimoto S., Yagi K., Wakasugi T., Takeda Y. (2013). Insulin autoimmune syndrome caused by an adhesive skin patch containing loxoprofen-sodium. *Internal Medicine*.

[34] Kamei S., Kaneto H., Shigemoto R. (2016). Human serum albumin: possible cause of insulin autoimmune syndrome. *Journal of Diabetes Investigation*.

[35] Sheng X., Ye X., Shi X., Lu L., Lu A. (2020). Combinations of plasma exchange with steroids treats *α*-lipoic acid induced insulin autoimmune syndrome in a Chinese woman. *Endokrynologia Polska*.

[36] Balestrieri A., Magnani E., Ragazzini C., Pasini G. (2015). Primary insulin autoimmune syndrome in an Italian woman: a case report. *Italian Journal of Medicine*.

[37] Roh E., Kim Y. A., Ku E. J., Bae J. H., Kim H. M. (2013). Two cases of methimazole-induced insulin autoimmune syndrome in graves’ disease. *Endocrinol Metab (Seoul)*.

[38] Su C. T., Lin Y. C. (2016). Hyperinsulinemic hypoglycemia associated with insulin antibodies caused by exogenous insulin analog. *Endocrinol Diabetes Metab Case Rep*.

[39] Kaneko K., Satake C., Izumi T., Tanaka M., Yamamoto J. (2019). Enhancement of postprandial endogenous insulin secretion rather than exogenous insulin injection ameliorated insulin antibody-induced unstable diabetes: a case report. *BMC Endocrine Disorders*.

[40] Torimoto K., Okada Y., Mori H., Tanaka Y. (2016). Two sisters with graves’ disease and similar clinical features who tested positive for anti-insulin antibodies after thiamazole treatment. *Internal Medicine*.

[41] Censi S., Albergoni M. P., Gallo N., Plebani M., Boscaro M., Betterle C. (2018). Insulin autoimmune syndrome (Hirata’s disease) in an Italian patient: a case report and review of the literature. *Clinical Chemistry and Laboratory Medicine*.

[42] Cappellani D., Macchia E., Falorni A., Marchetti P. (2020). Insulin autoimmune syndrome (Hirata disease): a comprehensive review fifty years after its first description. *Diabetes Metab Syndr Obes*.

[43] Dozio N., Scavini M., Beretta A. (1998). Imaging of the bufferingeffect of insulin antibodies in the autoimmune hypoglycemic syndrome. *Journal of Clinical Endocrinology and Metabolism*.

[44] Tinmanee R., Buranagan R., Ploybutr S., Lertwattanarak R., Sriwijitkamol A. (2017). Rare cause of recurrent hypoglycemia: insulin autoimmune syndrome. *Case Rep Endocrinol*.

[45] Lin S. D., Hsu S. R. (2019). Glucose changes in a patient with insulin autoimmune syndrome demonstrated by continuous glucose monitoring. *AACE Clin Case Rep*.

[46] Lee S. H., Oh S. H., Chung W. Y. (2013). Insulin autoimmune syndrome induced by methimazole in a Korean girl with Graves’ disease. *Annals of Pediatric Endocrinology & Metabolism*.

[47] Alam S., Ozair M., Ahmad J. (2016). Hypoglycemia due to Insulin Autoimmune Syndrome: a rare cause not to be forgotten. *Journal of Clinical and Translational Endocrinology: Case Reports*.

[48] Alrashidi E., Alessa T. (2019). Insulin autoimmune syndrome in a 25-year-old, previously healthy Kuwaiti man. *Case Rep Endocrinol*.

[49] Cappellani D., Sardella C., Campopiano M. C., Falorni A., Marchetti P. (2018). Spontaneously remitting insulin autoimmune syndrome in a patient taking alpha-lipoic acid. *Endocrinol Diabetes Metab Case Rep*.

[50] Basu A., John F. (2005). Insulin autoimmunity and hypoglycemia in seven white patients. *Endocrine Practice*.

[51] Jing K., Han Z., Junlin F. (2017). Preliminary investigation on diagnostic value of polyethylene glycol precipitation and gel filtration chromatography in insulin autoimmune syndrome. *Chinese Journal of Endocrinology and Metabolism*.

[52] Wong S. L., Priestman A., Holmes D. T. (2014). Recurrent hypoglycemia from insulin autoimmune syndrome. *Journal of General Internal Medicine*.

[53] Yuan T., Li J., Li M. (2019). Insulin autoimmune syndrome diagnosis and therapy in a single Chinese center. *Clinical Therapeutics*.

[54] Koga M., Inada S., Taniguchi J. (2017). High glycated albumin (GA) levels and the GA/HbA1c ratio in patients with insulin autoimmune syndrome. *Diabetology International*.

[55] Philippon M., Sejil S., Mugnier M., Rocher L., Guibergia C. (2014). Use of the continuous glucose monitoring system to treat insulin autoimmune syndrome: quantification of glucose excursions and evaluation of treatment efficacy. *Diabetic Medicine*.

[56] Boro H., Gupta U., Singh C., Malhotra R., Khadgawat R. (2021). Continuous glucose monitoring and Rituximab treatment in insulin autoimmune syndrome. *Diabetes & Metabolic Syndrome*.

[57] Lanas A., Paredes A., Espinosa C., Caamaño E., Pérez-Bravo F. (2015). Insulin autoimmune syndrome: report of two cases. *Revista Medica de Chile*.

[58] Boro H., Gupta U., Singh C., Malhotra R., Khadgawat R. (2020). Insulin autoimmune syndrome - a case series. *European Endocrinology*.

[59] Sun L., Fang W., Yi D., Sun W., Wang C. (2021). Analysis of the clinical characteristics of insulin autoimmune syndrome induced by methimazole. *Journal of Clinical Pharmacy and Therapeutics*.

[60] Yoshino H., Kawakami K., Kohriyama K., Yoshino G., Matsunaga S. (2020). Long-termfollow-up of insulin autoimmune syndrome in an elderly patient. *Clin Case Rep*.

[61] Oest L., Roden M., Müssig K. (2022). Comparison of patient characteristics between East Asian and non-East Asian patients with insulin autoimmune syndrome. *Clinical Endocrinology*.

[62] Batra C. M., Kumar K., Goyal M. (2021). Steroid-refractory insulin autoimmune syndrome treated with rituximab and continuous glucose monitoring. *Cureus*.

[63] Saxon D. R., McDermott M. T., Michels A. W. (2016). Novel management of insulin autoimmune syndrome with rituximab and continuous glucose monitoring. *Journal of Clinical Endocrinology and Metabolism*.

[64] Woo C. Y., Jeong J. Y., Jang J. E. (2015). Clinical features and causes of endogenous hyperinsulinemic hypoglycemia in Korea. *Diabetes & Metabolism Journal*.

